# Host-feeding patterns of *Aedes (Aedimorphus) vexans arabiensis*, a Rift Valley Fever virus vector in the Ferlo pastoral ecosystem of Senegal

**DOI:** 10.1371/journal.pone.0215194

**Published:** 2019-10-04

**Authors:** Biram Biteye, Assane Gueye Fall, Momar Talla Seck, Mamadou Ciss, Mariame Diop, Geoffrey Gimonneau

**Affiliations:** 1 Institut Sénégalais de Recherches Agricoles/Laboratoire National de l’Elevage et de Recherches Vétérinaires BP 2057 Dakar-Hann, Sénégal; 2 CIRAD, UMR INTERTRYP, Montpellier, France; 3 Centre International de Recherche–Développement sur l’Elevage en zone subhumide, Bobo-Dioulasso 01, Burkina Faso; 4 INTERTRYP, Univ Montpellier, CIRAD, IRD, Montpellier, France; Faculty of Science, Ain Shams University (ASU), EGYPT

## Abstract

**Background:**

Host-vector contact is a key factor in vectorial capacity assessment and thus the transmission of mosquito-borne viruses such as Rift Valley Fever (RVF), an emerging zoonotic disease of interest in West Africa. The knowledge of the host-feeding patterns of vector species constitutes a key element in the assessment of their epidemiological importance in a given environment. The aim of this work was to identify the blood meal origins of the mosquito *Aedes vexans arabiensis*, the main vector of RVF virus in the Ferlo pastoral ecosystem of Senegal.

**Methodology/principal findings:**

Engorged female mosquitoes were collected in Younouféré in the pastoral ecosystem in the Ferlo region during the 2014 rainy season. CO_2_-baited CDC light traps were set at six points for two consecutive nights every month from July to November. Domestic animals present around traps were identified and counted for each trapping session. Blood meal sources of engorged mosquitoes were identified using a vertebrate-specific multiplexed primer set based on cytochrome b. Blood meal sources were successfully identified for 319 out of 416 blood-fed females (76.68%), of which 163 (51.1%) were single meals, 146 (45.77%) mixed meals from two different hosts and 10 (3.13%) mixed meals from three different hosts. *Aedes vexans arabiensis* fed preferentially on mammals especially on horse compared to other hosts (FR = 46.83). Proportions of single and mixed meals showed significant temporal and spatial variations according to the availability of the hosts.

**Conclusion:**

*Aedes vexans arabiensis* shows an opportunistic feeding behavior depending on the host availability. This species fed preferentially on mammals especially on horses (primary hosts) and ruminants (secondary hosts).

## Introduction

Rift Valley Fever (RVF) is an emerging zoonotic vector-borne viral infection [1] considered as a major problem of public and veterinary health as evidenced by various outbreaks in Africa [2–6]. This disease causes significant economic gaps in terms of animal deaths and economic losses in the affected countries [7–9]. Mosquitoes of the genera *Aedes* and *Culex* are the main vectors of RVF virus (RVFV) and transmission mainly occurs during inter-epizootic periods [1]. RVF is endemic in Senegal, especially in the Ferlo region [10, 11]. The transmission of the virus is seasonal and caused by the mosquitoes *Aedes*. *(Aedimorphus) vexans arabiensis* (Patton) and *Culex*. *(Culex) poicilipes* (Theobald) with peaks of transmission at the end of the rainy season [12–14]. Disease control is difficult because mosquito vectors are able to fly on long distances and escape the border sanitary barriers. Moreover, vector control methods are not used to control RVF outbreaks because they are costly and difficult to implement and could have important environmental and ecological consequences. However, hosts such as cattle could be treated with an efficient insecticide against the bites of mosquitoes, or parked at night in a fence surrounded by impregnated net to reduce vectorial transmission in RVF outbreaks [15, 16].

The host-vector contact is a key factor in vectorial capacity assessment and the transmission of vector-borne pathogens. Understanding host-feeding pattern of vector species populations and its variation in space and time is important for a better knowledge of the role of these vectors in pathogens transmission, and thus in the design of accurate vector control measures or strategies [17]. Host choice is affected by innate preferences and environmental factors such as host diversity, density and distribution [18]. Although many studies on host preferences have been conducted for various mosquitoes, biting midges or tick vector species [17–22], so far in Senegal the molecular approach has been poorly used to identify the host blood meals of disease vectors. Earlier investigations [13, 19, 23] had used immunological assays that have several inherent problems such as efficiency and reliability of blood meal identification [22, 24]. The PCR based assays using different genetic markers have been developed for vectors targeting potential hosts (pigs, humans, goats, dogs, cows and avians) for malaria, West Nile (WN) fever, African Horse Sickness or bluetongue research purposes [17, 25–27]. The PCR-based technology using host mitochondrial DNA provides a more direct approach to the identification of host species and increases sensitivity and specificity [22]. Mitochondrial DNA, particularly the cytochrome b (Cyt b), has been used extensively in various studies [28–31] because it exhibits a high level of interspecific polymorphism which helps to design species specific primers [32]. In this study, we have used a vertebrate-specific multiplexed primer set based on Cyt b to identify the blood meal origins of engorged females of *Ae*. *v*. *arabiensis* caught during field collections. The aim of this work was to better understand the host-feeding patterns of RVFV vectors in the Ferlo pastoral ecosystem.

## Material and methods

### Study area

**Fig 1:**
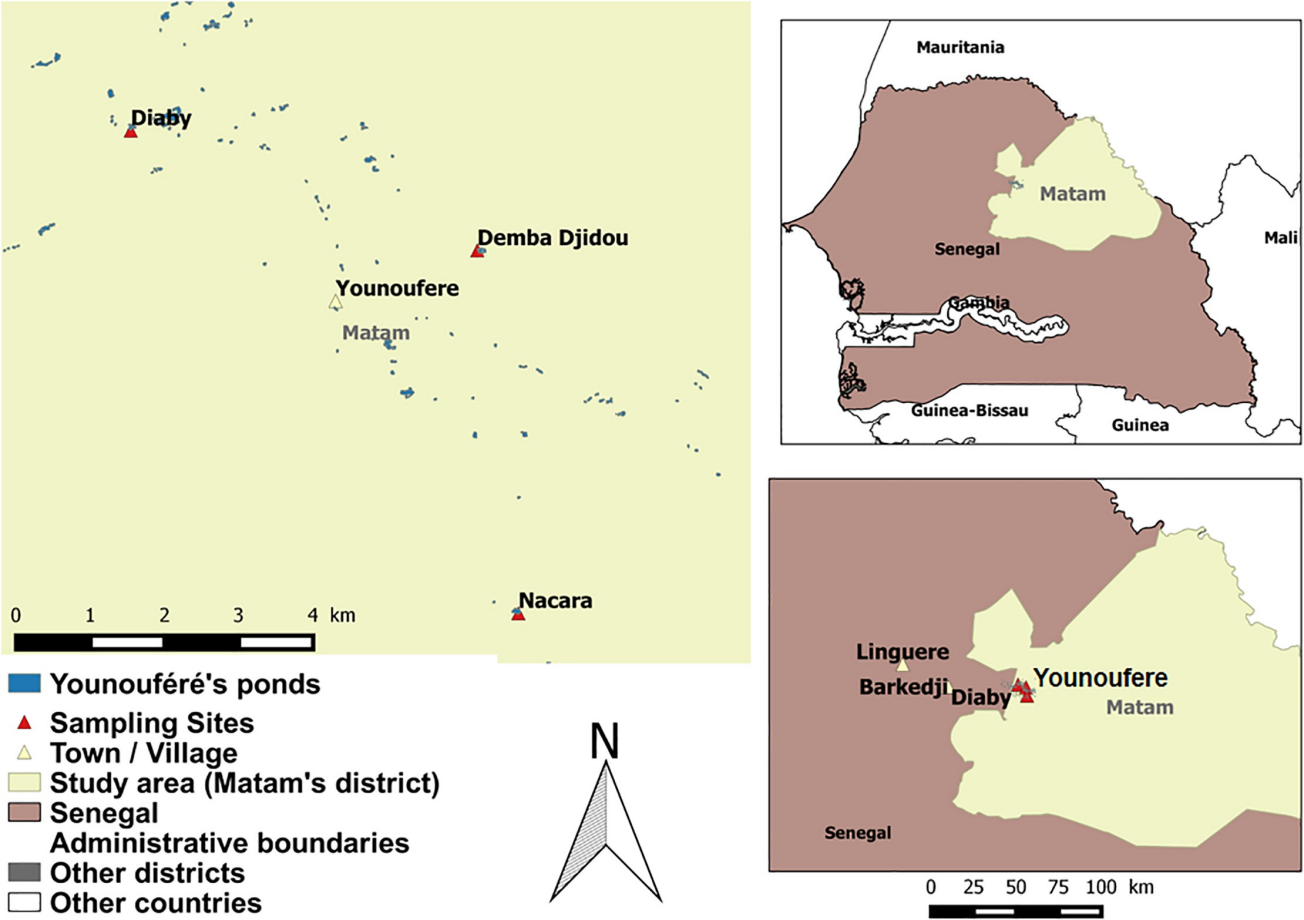
Location of the three sampling sites in Ferlo pastoral ecosystem (northern Senegal). Top-right corner figure: Senegal map and area of interest (Matam). Bottom-right corner figure: red triangles correspond to the three sampling sites, while the other ones represent main towns/villages near the sampling sites. Main figure: detail of the locations of the three sampling sites in Younouféré.

The study was performed around the Younouféré village (15°16'08.7''N and 14°27'52.5''W), a pastoral area located in the Ferlo region (central north of Senegal), during the 2014 rainy season. Younouféré is surrounded by small hamlets of which three were selected as sampling sites: Diaby (15°17'18.1''N, 14°29'07.9''W), Demba Djidou (15°16'53.6''N, 14°27'04.8''W) and Nacara (15°13'23.1''N, 14°26'18.8''W) (Fig 1). The area is characterized by a hot dry climate with a short rainy season (from June to October) and a long dry season (November to May), with mean annual rainfall ranging from 300 to 500 mm and a number of rainy days around 35.8 [33]. It is also characterized by a semi-arid steppe and many temporary ponds filled with rainfall and used by humans and animals as the main free sources of water during the rainy season [15, 34]. These ponds are the natural habitats of many species of birds, reptiles and rodents, and the breeding and resting sites for RVFV mosquito vectors. During the rainy season, the region becomes a high transhumance area where a high number of herds of domestic animals (cattle, sheep and goats) are concentrated around natural temporary ponds thus becoming risk areas due to the presence of vectors and the endemicity of the RVFV.

### Mosquito collection and survey of vertebrate hosts

Mosquito collection was conducted monthly from July to November 2014. During two consecutive nights, two traps (2 nights = 4 traps/site) were simultaneously set per site at about 1.5m heights from the ground: one close to a natural water point (ponds) and another close to a herd pen. Mosquitoes were trapped nightly (from 6 PM to 6 AM) using miniature CDC light traps (BioQuip # 2836Q-6VDC, Rancho Dominguez, USA) baited with CO_2_. Distances between water source and herd pen varied from 100 to 800m. In the field, the collected mosquitoes were killed by freezing in dry ice, sorted by genus on a chill table, put in 15 or 50 ml centrifuge tubes/cryo-tubes and transported in dry ice to the laboratory where they were identified according to sex and species on a chill table (-20°C) using morphological keys [35, 36] and identification software [37–39]. The *Ae*. *v*. *arabiensis* freshly engorged females were placed individually in eppendorf tubes (0.5ml) and stored at -20°C until the analysis of the origin of the blood meals by PCR. Information on vertebrate hosts’ diversity around each trapping site and their relative abundance was recorded monthly. The presence of thousands of temporary ponds also suggests a great diversity of wild fauna on which mosquito could feed. However, the choice of primers used in this study was guided by the hosts (domesticated animals, human and birds) identified around the sampling sites.

### Extraction of genomic DNA and PCR amplification

Genomic DNA was individually extracted using the modified Chelex resin 10% extraction protocol (Resin Chelex100 ^®^, Chelating Ion Exchange Resin, Bio-Rad, France) [40, 41]. Separated from the rest of the body, the mosquito's abdomen was ground with sand using piston in an eppendorf tube of 1.5 ml. After grinding, a volume of 500 μl of Chelex solution was added to each tube. The tubes were incubated at 56° C for 2 hours by vortexing every 30 minutes (min) and then at 95° C for 1 hour by vortexing every 20 min. Immediately after heating (thermal lysis), the tubes were centrifuged at 13,000 revs/min for 1 min to pellet the Chelex resin with inhibitor ions and cellular debris. The supernatants were gently transferred into new tubes and stored at -20° C until amplification of gene of interest. Molecular identification was based on the amplification of the cytochrome b region of blood DNA [17, 25, 26, 42, 43]. Two multiplex PCR were performed (Table 1) to separate cattle, sheep and goat first [17, 27] then dog and human [42]. Two simplex PCR were used (Table 1) to identify blood meal from horse [17, 27] and from bird [26].

**Table 1:** Primers set used for the identification of blood meal origin in mosquito abdomens.

Primers names	Primers sequences (5' →3')	Length (pb)
FP UNIV2	TGAGGACAAATATCATTYTGAGGRGC	-
RP OVIS (*Ovis artes*)	GGCGTGAATAGTACTAGTAGCATGAGGATGA	336
RP CAPRA (*Caprus hircus)*	TTAGAACAAGAATTAGTAGCATGGCG	313
RP BOS (*Bos taurus*)	TAAGATGTCCTTAATGGTATAGTAG	287
RP UNREV 1025	GGTTGTCCTCCAATTCATGTTA	-
FP Human 741(*Homo*. *sapiens)*	GGCTTACTTCTCTTCATTCTCTCCT	334
FP DOG 368 (*Canus lupus*. *familiaris*)	GGAATTGTACTATTATTCGCAACCAT	680
FP UNIV3	TTTTTTTTTTTTCGVTCHATYCCHAAYAAACTAGG	-
RP EQUUS (*Equus caballus*)	TACGTATGGGTGTTCCACTGGC	208
FP AVIAN	GACTGTGACAAAATCCCNTTCCA	-
RP AVIAN	GGTCTTCATCTYHGGYTTACAAGAC	508

**Abbreviations: RP;** Reverse Primer, FP; Forward Primer

### Statistical analysis

All night-traps allowed the collection of many specimens of mosquitoes of which a high number of engorged females of *Ae*. *v*. *arabiensis*. However, a subsample was performed for the identification of blood meals. Thus, for each month (from July to November) a maximum of 50 individuals were randomly selected from each trap point (3 ponds and 3 herd pens) which collection exceeded 50 engorged individuals. For those that did not reach 50 engorged individuals, all mosquitoes were analyzed. Non-parametric Kruskal-Wallis and Mann-Withney-U tests and the Chi-squared test were used to assess differences in blood meal proportions in time and between trap points. After descriptive analyses, the forage ratios (FR) were calculated [44–46] to provide a standard index of host selection for *Ae*. *v*. *arabiensis*. This FR was calculated for each host category as the percentage of positive blood meals divided by the percentage of the available hosts. FR > 1 indicating preference, FR < 1 indicating avoidance, and FR approaching 1 indicating little preference or avoidance [44, 45]. All of the analyses were carried out using R software [47].

## Results

The 60 night-traps (2 traps x 2 nights x 3 sites x 5 months) at 6 trap points (3 ponds and 3 herd pens) allowed the collection of 104,352 specimens of *Ae*. *v*. *arabiensis* of which 91,660 (87.84%) were unfed females, 2,150 (2.06%) were males and 10,542 (10.10%) were engorged females. The subsampling carried out allowed the selection of 416 engorged females (Table 2). Identification of blood meal sources was successful for 319 out of the 416 blood-fed females (76.68%), of which 163 (51.10%) were identified as single meals (from one species), 146 (45.77%) as mixed meals from two different host species and 10 (3.13%) as mixed meals from three different host species. The 97 (23.32%) remaining blood meals could not be determined with the primers used. The number and type of meal at each trapping point are shown in Table 2 and S1 Table. Of the 156 mixed meals, 141 (90.38%) involved horses; of which 131 came from two different hosts and 10 coming from three different hosts. Of the 146 dual mixed meals, 81 (55.48%) concerned both sheep and horses, 36 (24.66%) concerned both goats and horses and only 3 (2.05%) concerned both sheep and goats. Two out of 156 mixed meals (1.28%) concerned humans showing a very high zoophilic rate. Also 6 out of 163 single meals (3.68%) and 20 out of 156 mixed meals (12.82%) concerned birds, which represents a high mammophilic rate.

**Table 2:** Origin of blood meals taken by *Aedes vexans arabiensis* females collected at different sites.

Hosts	Diaby camp	Diaby Pond	Djidou Camp	Djidou Pond	Nacara Camp	Nacara Pond	Total
Goat	12	7	3	9	5	9	45
Cattle	4	5	0	4	1	3	17
Sheep	9	4	1	1	3	4	22
Human	0	0	0	0	0	0	0
Dog	0	0	0	0	0	0	0
Horse	22	10	15	10	8	8	73
Bird	1	1	1	2	0	1	6
Goat-Sheep	0	1	0	1	0	1	3
Goat-Cattle	0	0	0	1	0	0	1
Goat-Horse	18	1	5	4	4	4	36
Goat-Bird	0	0	0	1	0	2	3
Sheep-Horse	53	4	13	1	6	4	81
Sheep-Bird	3	1	0	0	0	1	5
Cattle-Horse	3	1	0	1	1	4	10
Cattle-Bird	0	2	0	0	0	1	3
Human-Horse	0	0	1	0	1	0	2
Horse-Bird	0	1	1	0	0	0	2
Goat-Sheep-Horse	1	0	0	0	0	0	1
Goat-Cattle-Horse	0	0	0	1	0	0	1
Goat-Dog-Horse	0	0	0	0	0	1	1
Goat-Horse-Bird	1	0	0	0	0	1	2
Sheep-Horse-Bird	3	0	0	1	0	0	4
Cattle-Horse-Bird	1	0	0	0	0	0	1
Unidentified	27	17	17	12	18	6	97
Total	158	55	57	49	47	50	416

Abbreviation: Camp; livestock pen

**Fig 2:**
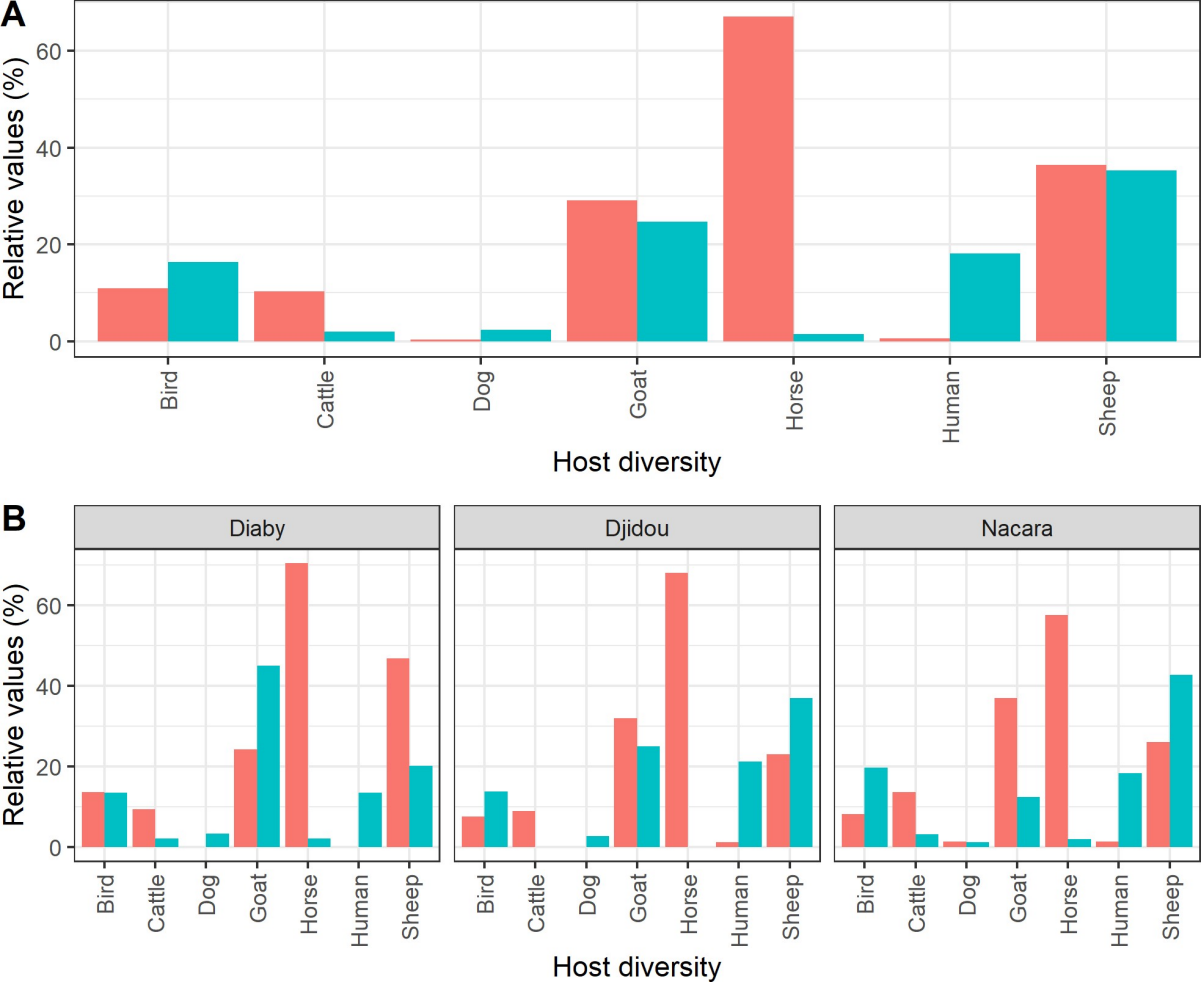
Origin of blood meals (relative values) taken by *Ae*. *vexans arabiensis* females collected at different sites (in pink) and vertebrate host proportions around trapping site (in cyan blue). (A) represents the compiled frequencies of all study sites and (B) represents the corresponding frequencies according to study sites.

*Aedes v*. *arabiensis* fed preferentially on horse (FR = 46.83) and cattle (FR = 5.16), than goat, sheep, human and bird (Table 3; Fig 2). Table 3 shows the compiled FR results of the three sampling sites (Diaby, Djidou and Nacara).

**Table 3:** *Aedes v*. *arabiensis* forage ratios for domestic animals, human and bird in all study sites.

Hosts	% Host (n = 349)	% Blood meals (n = 319)	Forage Ratios
**Bird**	16.33%	10.97%	0.67
**Cattle**	2.01%	10.34%	5.16
**Dog**	2.29%	0.31%	0.14
**Goat**	24.64%	29.15%	1.18
**Horse**	1.43%	67.08%	46.83
**Human**	18.05%	0.62%	0.03
**Sheep**	35.24%	36.36%	1.03

The number of meals (altogether) taken on the different hosts throughout the study months are presented in Table 4. No blood fed mosquitoes were caught in November. The calculated proportions of single blood meals and mixed blood meals varied significantly over the study period (χ^2^ = 258.1; *df* = 3, P < 0.001 and χ^2^ = 83.34; *df* = 3, P < 0.001, respectively); and between trapping points (χ^2^ = 21.9; *df* = 5, P < 0.001 and χ^2^ = 153.1; *df* = 5, P < 0.001, respectively). The highest abundances of single meals (n = 129/163; 79.14%) and mixed meals (n = 84/156; 53.85%) were observed in August.

**Table 4:** Variation of blood meals number taken on hosts along the study period.

Month	Total	Single meals (%)							Mixed meals (%)	Unidentified (%)
		Goat	Sheep	Cattle	Human	Dog	Horse	Bird		
July	**7**	2 (28.57)	0 (0)	0 (0)	0 (0)	0 (0)	0 (0)	0 (0)	5 (71.43)	0 (0)
August	**294**	37 (12.59)	13 (4.42)	13 (4.42)	0 (0)	0 (0)	60 (20.41)	6 (2.04)	84 (28.57)	81 (27.55)
September	**56**	3 (5.36)	4 (7.14)	3 (5.36)	0 (0)	0 (0)	7 (12.5)	0 (0)	31 (55.36)	8 (14.29)
October	**59**	3 (5.09)	5 (8.47)	1 (1.69)	0 (0)	0 (0)	6 (10.17)	0 (0)	36 (61.02)	8 (13.56)
November	**0**	0 (0)	0 (0)	0 (0)	0 (0)	0 (0)	0 (0)	0 (0)	0 (0)	0 (0)
Total	**416**	45 (10.81)	22 (5.28)	17 (4.1)	0 (0)	0 (0)	73 (17.55)	6 (1.44)	156 (37.5)	97 (23.32)

## Discussion

The identification of the blood meals of hematophagous arthropods is very important in determining the host-vector contact in nature. The PCR-based technology using host mitochondrial DNA has been used to eliminate some constraints of immunological assays [22] and to provide a more direct and sensitive approach to identify host species because sera do not have to be collected and specific antibodies produced [17]. In our study, the origin of the blood meal was successfully identified in 76.68% of the cases. Similar rates have been observed in several studies [25, 48–50], while others [43, 51] got lower rates. These different observations would probably be explained by the quantity and quality of the blood (partially digested or not), the diagnostic techniques, the range of primers used compared to the domesticated and/or wild fauna of the localities. Of the 319 meals identified, 156 (48.9%) were mixed meals and were mostly taken in August (53.85%). Similar observations have already been made by Ba *et al*. [19] and Fall *et al*. [23]. The high rate of mixed meals could be explained by the peak abundance of *Ae*. *v*. *arabiensis* in August [52] and also by the scarcity of hosts [23]. Indeed, abundance of mosquitoes leads to a greater nuisance and consequently of a self-defense reflex in hosts. These self-defense reflexes increase the rate of interrupted blood meals, therefore favoring shifts between host species [23]. Thus, August seems to be the most favorable period for pathogens transmission between hosts by *Ae*. *v*. *arabiensis*.

Our results showed an opportunistic zoophagous behavior of *Ae*. *v*. *arabiensis* populations that could feed on at least seven different hosts. However *Ae*. *v*. *arabiensis* showed a significant preference to feed on mammals and especially on horses (FR = 46.83). Such tendency to feed on horses has already been observed in previous studies [13, 19, 23] making this mosquito a potential bridge vector of the West Nile (WN) virus in the area [23]. Studies in Mexico and the northern United States (USA) also confirmed *Ae*. *vexans’* preference for mammalian vertebrates [50, 53]. Molaei & Andreadis [53] showed that populations of *Ae*. *vexans* mainly fed on deer (80%) and rarely on the horses (9.2%). This was mainly due to the fact that deer populations were more abundant and available than equine populations or simply by an acquired preference for deers. In our case, the large percentage of meals taken on horse is mainly explained by the fact that these animals are usually found at night in the immediate vicinity of the ponds where they find water and pasture and are the first available animals for active females for their blood meal. Therefore, the predominance of meals on horses could reflect a greater availability of the horses rather than a trophic preference compared to cattle and sheep.

*Aedes v*. *arabiensis* fed secondarily on ruminants (cattle, goat and sheep), rarely on birds, humans and dogs, confirming the previous observations [13, 19, 23, 54] and the important role that domestic ruminants probably play in the epidemiology of RVF. Therefore, the risk of transmission of the RVFV would be high in this endemic area [11] with a high host vector contact, leading to the occurrence or resurgence of the disease. Most of the authors [23, 50, 53] showed a moderate or low rate of blood meal (0.4 to 10%) of *Ae*. *v*. *arabiensis* on birds. It should also be noted that most of the mixed meals (mainly recorded in August) originated from "horses-sheep" (55.48%) and "horses-goats" (24.66%). This finding is in line with the assumption that domestic ruminants probably play a more important role in the epidemiology of RVF than the other hosts since horses do not develop high viremia and are resistant to RVFV infection. The high rate of unidentified meals (23.32%) could probably be explained by the quantity [51] and quality of the blood [55] probably due to the gradual intensification of the vector digestive gland functions (blood partially digested or not) [51, 56, 57], the diagnostic techniques, or the range of primers used compared to the domesticated and/or wild fauna (rodents, reptiles, lagomorphs etc.) present in these localities (ponds) and which have not been tested in this study.

## Conclusion

The blood meal sources of *Ae*. *v*. *arabiensis* varied according to host availability and trap points and confirmed the opportunistic feeding behavior of this mosquito species. In the Ferlo pastoral ecosystem, domestic ruminants probably play a more important role in the epidemiology of RVF than the other hosts.

### Additionnal information

#### Ethics statement

No specific permits were required for the described field studies: a) no specific permissions were required for these locations/activities: collections from private hamlets were carried out with the consent of the owners; b) ponds are not privately-owned or protected; c) the field studies did not involve endangered or protected species. They do not involve the use of personal data collection or processing, or the use of animals, nor elements that may cause harm to the environment, to animals or plants nor dual-use items. Ethical approval was not required because the study was conducted as part of the national epidemiological surveillance system for national notifiable diseases.

## Supporting information

S1 Table Abundance, host diversity and blood meal results for *Ae*. *vexans arabiensis* according to study sites. (CSV)
